# Impact of Magnetite Nanoparticles Coated with Aspartic Acid on the Growth, Antioxidant Enzymes Activity and Chlorophyll Content of Maize

**DOI:** 10.3390/antiox11061193

**Published:** 2022-06-17

**Authors:** Mihaela Răcuciu, Andreea Tecucianu, Simona Oancea

**Affiliations:** 1 Environmental Sciences and Physics Department, Faculty of Sciences, Lucian Blaga University of Sibiu, Dr. I. Ratiu Street, No. 5–7, 550012 Sibiu, Romania; 2 Dacia Plant Company, Harmanului Str., Bot, 507015 Brașov, Romania; s_andreea_cristina@yahoo.com; 3 Agricultural Sciences and Food Engineering Department, Lucian Blaga University of Sibiu, Dr. I. Ratiu Street, No. 7–9, 550012 Sibiu, Romania; simona.oancea@ulbsibiu.ro

**Keywords:** magnetite nanoparticles, maize, plant growth, catalase, peroxidase, chlorophylls

## Abstract

In recent decades, magnetite nanoparticles received greater attention in nanobiotechnology due to wide applications. This study presents the influence of the oxidative stress caused by magnetite nanoparticles coated with aspartic acid (A-MNP) of 9.17 nm mean diameter size, on maize (*Zea mays)* seedlings, in terms of growth, enzymatic activity and chlorophyll content as evaluated in exposed plant tissues. Diluted suspensions of colloidal magnetite nanoparticles stabilized in water were added to the culture medium of maize seeds, such as to equate nanoparticle concentrations varying from 0.55 mg/L to 11 mg/L. The obtained results showed that the growth of maize was stimulated by increasing the level of A-MNPs. Plant samples treated with different concentrations of A-MNP proved increased activities of catalase and peroxidase, and chlorophyll content, as well. The exposure of plants to magnetite nanoparticles may induce oxidative stress, which activates the plant defense/antioxidant mechanisms.

## 1. Introduction

Large amounts of reactive oxygen species (ROS) can cause cellular damages in plants exposed to different stress conditions. For several decades, iron oxide nanoparticles received more attention within nanobiotechnology research, being widely used in various fields of science. Among different types of iron oxide nanoparticles, magnetite (Fe_3_O_4_) nanoparticles are successfully used in biomedical applications, exhibiting low toxicity and high stability [1]. Recently, nanobiotechnology has extended its relevance in plant science and agriculture [2,3]. There is a particular interest in the applications of magnetic nanoparticles to plants for agricultural and horticultural benefits. Moreover, the production and availability of magnetic nanoparticles have been increasing considerably, extending the possibility of releasing them into the environment, including by accident. Therefore, monitoring the nanotoxicity of magnetic nanoparticles is important for evaluating the risk to human health and the environment [4]. The magnetic nanoparticles nanotoxicity and their potential accumulation in plant organisms has become one of the current research topics in the context of considering the impact of engineered iron oxide nanoparticles, such as magnetite, on biotic environmental components [5]. Being an important unit of the ecosystem, plants provide a potential carrier for the transport of magnetic nanoparticles into the environment, going as far as their bioaccumulation in the food chain [6]. In plants, iron plays an important role in the biosynthesis of chlorophylls, photosynthesis and the respiration processes [7]. As a result, magnetic nanoparticles have an important role in germination, efficient growth and increasing yield [8]. Magnetic nanoparticles showed efficiency as nanofertilizers used to improve the accessibility of iron to plants and to control the function of some antioxidant enzymes and phytohormones, resulting in enhanced plant biomass accumulation, height and root length [9]. Recently, the development of biocompatible nanoparticles that have antioxidant properties has attracted a great deal of attention [10]. In addition, magnetite nanoparticles possess an intrinsic enzyme mimetic activity similar to natural peroxidase activity [11]. The peroxidase-like activity of magnetite nanoparticles is based on the coexistence of Fe^2+^/Fe^3+^ ion pairing in the structure, such as Fenton’s reagent, which are known to catalyze the breakdown of hydrogen peroxide [12]. Furthermore, nanocomposites containing iron oxides were suggested to be used against iron deficiency, with potential utilization in food science [13,14]. Magnetic nanoparticles have also been applied in the food industry for the detection of foodborne pathogens or toxins [15,16]. Therefore, due to the vast utilization of iron oxide nanoparticles in nanobiotechnology, the evaluation of their biocompatibility becomes important. Few studies evaluated the potential nanotoxicity of iron oxide nanoparticles on plants [8,17]. Many studies are focused on cellular responses such as seeds germination, proliferation and differentiation process, after exposure to iron oxide nanoparticles, showing both inhibitory and stimulatory effects [18,19]. Genotoxicity is one of the most disturbing effects induced by iron oxide nanoparticles on plants [20,21]. Nevertheless, reduced progress has been made in relation to understanding the impact of magnetic nanomaterials at the molecular level, which is an important step in estimating the possible mechanism of observed effects of nanoparticles on plants. Nanotoxicity mechanisms still remain unknown, but it could be assumed they are closely related to the chemical composition, chemical structure, size and surface area of nanoparticles.

This study aimed to reveal the plant growth indices, activity of antioxidant enzymes and chlorophyll content after the treatment of maize seedlings with magnetite nanoparticles coated with aspartic acid (A-MNP). Maize (*Zea mays*) is one of the three most important food crops worldwide, having an important role in the food security of humankind. The assessment of the phytotoxicity of nanoparticles on agriculturally important crop plants is crucial to human health. It has been confirmed that magnetite nanoparticles exhibited high antioxidant ability [22]. In addition, it has been found that amino acids, such as aspartic acid, used as a coating agent for the present investigated nanoparticles sample may also have specific antioxidant activity [23,24]. On the other hand, some research studies revealed that supplementation of plant seeds with essential amino acids is beneficial due to their nutritive value [25,26].

In our previous studies, magnetic nanoparticles coated with aspartic acid (A-MNP) were prepared and tested, showing great potential for biomedical applications. Therefore, starting from these results reported by Răcuciu et al. (2022) [27] regarding the synthesis and characterization of A-MNP and the study on the genotoxicity of a similar A-MNP sample, reported by Răcuciu (2020) [28], we continued the research as presented in this paper dealing with the evaluation of the A-MNP influence on maize seeds. To our knowledge, no results regarding the influence of magnetic nanoparticles coated with aspartic acid on plants have been reported. Considering this, the obtained results may be further useful in estimating the possible interaction mechanisms of magnetic nanoparticles with plant organisms.

## 2. Materials and Methods

### 2.1. Experimental Design

Maize seeds (*Zea mays*) were chosen as biological material because of its economic relevance in agriculture and food industry fields. The experimental lot of undamaged maize (*Zea mays)* seeds with uniform genetic features was provided by a local farmer from Săliște, Sibiu county, România, with the local maize being own-sourced. Forty seeds of each sample were let to germinate in petri dishes on moistened filter paper with 15 mL aqueous solutions of A-MNP with different volume fractions, in a controlled laboratory room at 24 °C, in darkness. Six different volume fractions of A-MNP aqueous suspensions (20, 40, 80, 160, 320 and 400 µL/L) were prepared for plant samples’ treatment. Selection of concentrations for the treatment was done following similar studies’ values for volume fractions of nanoparticles aqueous suspensions. For preparing A-MNPs aqueous suspensions, a native magnetite coated with aspartic acid (C_4_H_7_NO_4_) sample synthesized in our laboratory by an adapted controlled chemical precipitation approach at room temperature, was used. The native magnetite nanoparticles sample is suitable for biomedical applications due to its small particles, narrow size distribution, mean physical diameter of about 9.17 nm and good stability [27]. The A-MNP concentrations corresponding to the six volume fractions used in this experiment are shown in Table 1.

The control sample was let to germinate under the same environmental conditions, but the substrate was only supplied with distilled water (15 mL). A daily supply of every sample of *Zea mays* plantlets with 12 mL A-MNP aqueous suspension of a certain concentration was carried out for 7 days, after germination. Maize seedlings were grown under controlled conditions of environmental temperature (22.0 ± 0.5 °C), illumination (light/dark cycle: 14 h/10 h) and 70% humidity, in a laboratory climate room. The control samples were let to grow under the same environmental conditions and plants were supplied with only the same amount of deionized water.

### 2.2. Assay of Enzymes Activity

Catalase activity (CAT) was determined in maize extracts according to the method described by Luck (1965) [29]. The extract was added to 3 mL of H_2_O_2_-phosphate buffer and the decrease of absorbance was measured at 240 nm. The Specord 200 Plus UV–VIS spectrophotometer (Analytik Jena, Jena, Germany) was used. The enzyme activity was expressed in terms of units/assay, one unit being defined as the amount of enzyme required to decrease the absorbance by 0.05.

Peroxidase activity (POD) was determined in maize extracts according to the method described by Reddy et al. (1995) [30]. The extract was added to 3 mL of 0.05 M pyrogallol and 0.5 mL H_2_O_2_. The change in absorbance was measured at 430 nm. The Specord 200 Plus UV–VIS spectrophotometer (Analytik Jena, Jena, Germany) was used. The enzyme activity was expressed in terms of units, one unit being defined as the change in absorbance/minute at 430 nm.

### 2.3. Assay of Magnetite Nanoparticles Phytotoxicity

The A-MNP effects on chlorophyll levels for different nanoparticle concentrations in the culture media were determined. In the presence of low amounts of MgCO_3_ and CaCO_3_, fresh tissue (about 0.1 g) from each sample was crushed and homogenized in 5 mL of 90% acetone (from SILAL Trading, Chemical Reagent Company, Romania). The homogenate was centrifuged and filtrated. The pigments extract was supplemented with 90% acetone to 10 mL. The chlorophylls level in the fresh tissue was assayed by spectrophotometric method using a Specord 200 Plus UV–VIS spectrophotometer device (Analytik Jena, Germany) provided with quartz cells of 1 cm width. Spectrophotometric measurements were accomplished at the wavelengths of: 630 nm, 647 nm, 664 nm and 691 nm. The level of chlorophylls was calculated according to Ritchie’s Formulas (1)–(3) [31] and expressed in mg per gram fresh weight (mg/g):

Chl a = −0.3319 A630 − 1.7485 A647 + 11.9442 A664 − 1.4306 A691 (±0.0020) (1)

Chl b = −1.2825 A630 − 19.8839 A647 − 4.8860 A664 − 2.3416 A691 (±0.0076) (2)

Total Chl = 21.3877 A630 + 10.3739 A647 + 5.3805 A664 + 5.5309 A691 (±0.0056) (3)

In addition, the chlorophyll stability index (CSI) was calculated following the Formula (4) according to Sairam et al. (1997) [32]. CSI is an indicator used for the plant tolerance against stress conditions.

CSI = (total chlorophyll of treated sample ÷ total chlorophyll of control sample) × 100 (4)

Germination percentage (GP) was calculated according to the relationship (5) proposed by Dehnavi et al. (2020) [33]:

GP = (number of germinated seeds ÷ total number of seeds used per sample) × 100 (5)

The length of individual seedlings was measured after 7 days of growth with 1 mm precision using a simple ruler. The moisture content of fresh tissue samples was analyzed at 105 °C using a MAC210 infrared thermo-balance with 10^−3^% accuracy.

### 2.4. Statistical Analysis

Forty healthy seeds with uniform genes pool represented each experimental sample. For each batch of test seeds, the average plant lengths and the standard deviations (SD) were calculated. Using the Student *t*-test for the confidence level *P* = 95%, the confidence interval for every batch of plantlets was also evaluated.

All analyses were done in triplicate, using a specific weight from entire fresh green masses obtained for each experimental sample. The results were presented as mean ± SD (standard deviation). *p* < 0.05 was considered to be a significant difference. All statistical analyses and graph representation were performed using the Origin 64 and Microsoft Excel2016 software.

## 3. Results

**Figure 1:**
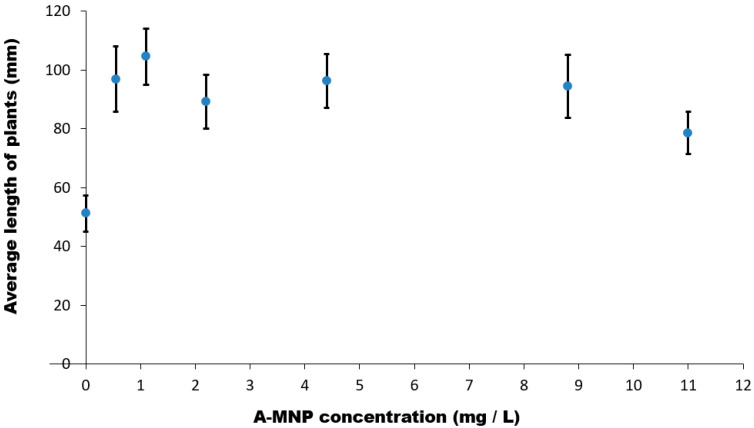
The average length of seedlings versus concentrations of A-MNPs. All average values are statistically significant.

The investigated A-MNP treatments positively influenced the shoot length of maize plants during the early ontogenetic stages. A stimulatory effect of increased concentrations of A-MNPs aqueous suspensions on the maize seedlings growth was recorded. Figure 1 shows the average length of maize seedlings versus six different concentrations of A-MNPs aqueous suspensions.

An over 50% increase of average seedlings length compared to the control was evidenced in the maize sample corresponding to A-MNP concentration of 1.1 mg/L, whereas for A-MNP concentrations higher than 1.1 mg/L, the stimulatory effect diminished gradually, reaching a 35% increase compared to the control. Statistically significant differences were revealed for all investigated A-MNP concentrations. By increasing the A-MNP concentration, the germination percentage (GP) decreased up to 18% in samples treated with 8.8 mg/L A-MNP compared to that of the control (GP = 85%). The A-MNP treatment of maize seeds seems to determine an increased moisture in the green tissue of young plants, up to 89.57% compared to that of the control (88.5%).

**Figure 2:**
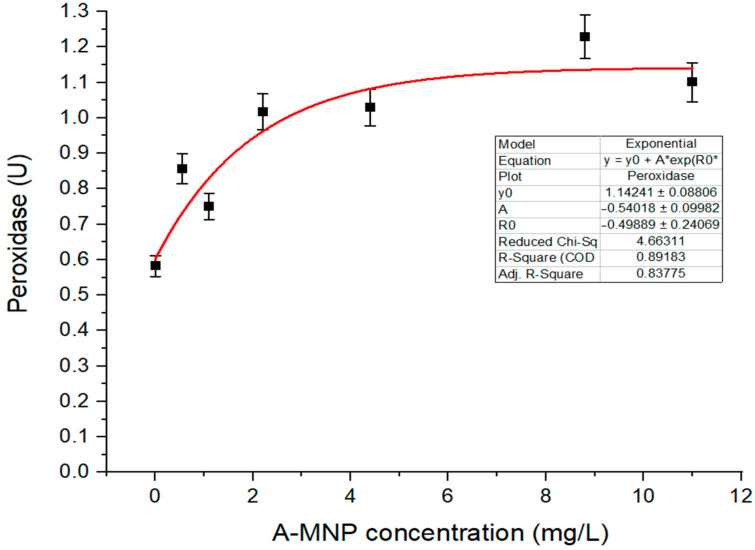
Peroxidase level versus concentrations of A-MNPs. All values are statistically significant in relationship with control. Graph fitting is realized with Origin64 software.

The A-MNP treatment did not show any inhibitory effects on POD activity of the maize leaves in contrast with the control (Figure 2). The enhanced level of POD activity favorizes the elimination of the excess ROS.

**Figure 3:**
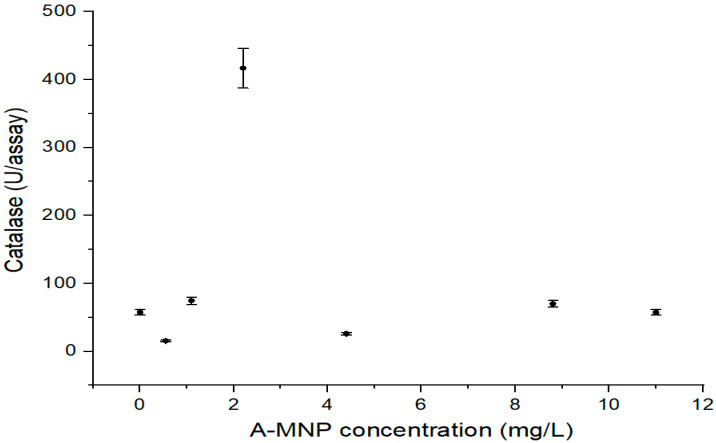
Catalase level versus concentrations of A-MNPs. All values are statistically significant in relationship with control.

Compared to control, the CAT activity of maize leaves was not inhibited by A-MNP treatment (Figure 3), with the exception of the sample treated with 0.55 and 4.4 mg/L A-MNP, respectively. A significant increase of CAT activity was observed in the sample treated with 2.2 mg/L A-MNP (7.25 times higher than control).

**Figure 4:**
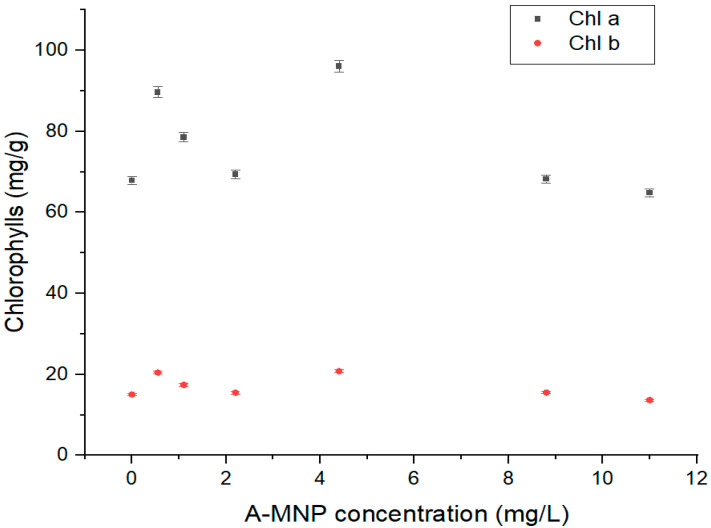
Evolution of the chlorophylls content with A-MNP concentration in maize plantlets (Chl a—chlorophyll a, Chl b—chlorophyll b).

According to the graphical plot of chlorophyll, as indicated in Figure 4, the level of the photosynthesis pigment increased in samples treated with lower A-MNP concentrations of 0.55–4.4 mg/L (up to 41% increasing) compared to that of the control sample (*p* < 0.05). For relatively high A-MNP suspension concentrations (8.8 and 11 mg/ L), the chlorophyll a level did not significantly vary compared to that of the control sample. A similar response was registered in the case of chlorophyll b content, which increased up to 38% for low A-MNP concentrations 0.55–4.4 mg/L.

A similar variation trend was noticed for the calculated total content of assimilatory pigments as that observed for chlorophylls a and b levels. Regarding the chlorophylls ratio (chlorophyll a/chlorophyll b), no statistically significant difference was found in relation to the control sample, whereas the A-MNP concentration was raised from 0.55 mg/L to about 11 mg/L. In addition, the experimental results revealed a significantly higher chlorophyll stability index (CSI) for low A-MNP concentrations (between 0.55 and 4.4 mg/L), and a lower CSI for higher A-MNP concentrations (8.8 and 11 mg/L). The highest CSI value resulted in samples treated with 4.4 mg/L A-MNP, treatment which led to increased Chl a, Chl b, total assimilatory pigments content and CSIs of ~41%, 38%, 37% and 37%, respectively.

## 4. Discussion

Magnetic nanoparticles are known to cause oxidative stress due to variation of iron levels [34]. Iron plays an essential role in plant development and biological processes, e.g., photosynthesis, respiration and redox reactions [9]. Plant iron deficiency determines a decrease of the chlorophyll content and photosynthesis efficiency. It has been shown that iron oxide nanoparticles have the potential to improve the capacity of plants to absorb nutrients through interactions at the molecular level [35]. Being an important component of the cell redox systems, iron acts as a cofactor of various antioxidant enzymes such as catalase (CAT) and peroxidase (POD). Nanoparticles can cause oxidative stress through ROS production, and thus induce an antioxidant defense system in plants. Within the antioxidant plant defense systems, CAT and POD are considered important enzymes to counter hydrogen peroxide produced under stress conditions. In recent years, numerous scientific papers reported that the iron containing nanoparticles may be used to work out the iron deficiency in plants [13,14]. As an enzyme cofactor, iron is required for photosynthetic reactions being a key factor for the plant growth. Rui et al. (2016) showed the positive effect of iron oxide nanoparticles on the iron deficiency-sensitive plant, the peanut [9]. It was reported that nanoparticles could enter into roots through the apoplastic or symplastic route [36]. As claimed by Nel et al. (2006), the small nanoparticles size, their large surface area and capacity to produce ROS all play an important role in nanoparticles’ toxicity [37]. The presence of nanoparticles on the root surface can change their surface chemistry and consequently affect the uptake of nutrients [38]. In addition, some studies have shown that iron oxide nanoparticles increased the iron content of different parts of the plant [36,39].

Our results showed that the A-MNP added to the maize seedlings culture medium stimulated the antioxidant enzyme activity (CAT and POD), plant development and chlorophyll biosynthesis as well. Magnetite nanoparticles induced an antioxidant defense in maize plants. Pintilie et al. (2006) reported increased CAT activity for maize seedlings treated with different volume fractions of water suspensions of magnetic nanoparticles coated with citric acid, up to 100 mL/L [40]. Iannone et al. (2021) revealed increased CAT activity in soybean and alfalfa shoots treated with 50 and 100 mg/L magnetite nanoparticles coated with citric acid [41]. According to Wang et al. (2011), the CAT activity in ryegrass and pumpkin shoots increased significantly when treated with both 30 and 100 mg/L levels of magnetite nanoparticles 25 nm in size coated with polyvinylpyrrolidone [42]. Similarly, Hu et al. (2017) revealed increased CAT activity compared to that of control, for *Citrus maxima* shoots under magnetite nanoparticles treatment at concentrations between 20–100 mg/L [43]. In addition, the POD activities of *Citrus maxima* shoots treated with magnetic nanoparticles at concentrations of 20 and 100 mg/L were significantly higher than those of the control [43]. Li et al. (2013) reported that the presence of iron oxide nanoparticles (with 9 nm nanoparticle size and concentrations between 2 mg/L and 50 mg/L) resulted in significantly higher CAT and POD activities than those of the control, and increased seedling growth indices in watermelon plants [44]. Compared to other reported studies, our results on maize seedlings samples treated with relatively low concentrations of magnetite nanoparticles indicated no significant influence on photosynthesis, but a remarkable increase of plant length and chlorophyll content. The investigated levels of added A-MNP up to 11 mg/L affected the seedlings length, revealing a stimulatory effect on the maize seedlings growth. An important number of studies revealed stimulatory effects of magnetite nanoparticles on different plant species development. Thus, the results of Plaksenkova et al. (2019) revealed significantly increased shoot and root lengths in garden rockets exposed to nanoparticles at concentrations of 1, 2 and 4 mg/L, for five weeks [45]. Elfeky et al. (2013) showed stimulatory effects of iron oxide nanoparticles up to 3 mg/L on the growth of *Ocimum basilicum* on seedlings length, total plant mass and weight [46]. Similar data were obtained by Zahra et al. (2015), such as shoot and root elongation of *Lactuca sativa* after exposure to magnetite nanoparticles [47]. Kokina et al. (2020) showed that enhanced yellow medick seedlings length for magnetite nanoparticles added up to 4 mg/L [48]. The growth of barley seedlings was improved when exposed to 17 mg/L or up to 250 mg/L magnetite nanoparticles [36,49]. Magnetite nanoparticles (20 nm in size and concentrations between 20 and 50 mg/L) significantly promoted growth and increased chlorophyll content in *Pseudostellaria heterophylla* plants [50]. Enhanced growth, biomass and chlorophyll content were obtained for *Cannabis sativa* plants treated with 17 nm of magnetite nanoparticles at concentrations between 50 and 500 mg/L [51]. Chlorophyll levels increased in soybean and alfalfa seedlings treated with 50 and 100 mg/L magnetite nanoparticles coated with citric acid [41]. As shown in our previous work, magnetite nanoparticles of 8.9 nm, synthetized using tartaric acid as coating agent, exhibited positive effects on growth and the chlorophyll content of maize when added in concentrations of 11 mg/L to the seed medium [52].

The increase in the chlorophyll content after treatment with nanoparticles might be related to an increased iron level given by the magnetite nanoparticles. It has been reported that the release of iron from magnetite nanoparticles provides a continuous iron supply for the formation of photosynthetic protein complexes, which could contribute to plant growth [41]. A similar response was observed for both chlorophyll a and b; therefore, the chlorophyll ratio (chlorophyll a/chlorophyll b) was approximately constant for A-MNP concentrations ranging from 0.55 to 11 mg/L. The highest variation of chlorophyll ratio was about 5% for the highest A-MNP concentration, but not statistically significant. The chlorophyll ratio might be used as an indirect indicator of the photosynthesis process efficiency [52]. From this point of view, no significantly influence of A-MNP on the photosynthesis process at maize seedlings was noticed during our experiment. Al-Amri et al. (2020) reported that iron oxide nanoparticles of different sizes are effective in increasing the iron content of wheat plants [53]. In addition, the iron ions released by magnetite nanoparticles can assist as a cofactor of various antioxidant enzymes such as catalase (CAT) and peroxidase (POD), or cytochromes of the electron transport chain. Other samples of A-MNP which are similar to the hereby synthetized ones but using another protocol, were evaluated for their genotoxic effect on the maize root tip cells indicating that the mitotic division of cells was stimulated by nanoparticles with a low rate of aberrant cell occurrence [28]. Magnetic nanoparticles toxicity can arise in different ways: through toxicity of the precursors used for magnetic nanoparticles synthesis and through disturbances of the ongoing cellular mechanisms inside the vegetal tissue. For in vivo applications, magnetic nanoparticles should be nontoxic and biocompatible. The toxicity of magnetic nanoparticles depends on numerous factors, such as size, shape, structure, surface modification, concentration, dosage, biodistribution and biocompatibility [54]. Nanoparticles’ toxicity towards plants is species-dependent [55], being linked to the applied treatment (nanoparticle type and concentration) and to growth conditions. The A-MNP sample, used in this study, is suitable for biomedical applications due to dominant small particles with a narrow size distribution and a physical diameter of about 9.17 nm, showing good stability [27].

The present investigation confirmed a positive influence of A-MNP on the performance of maize seedlings. It seems that magnetite nanoparticles coated with aspartic acid, at 9.17 nm in size and at concentrations of 0.55–11 mg/L, have a low toxicity on maize seedlings.

## 5. Conclusions

The addition of low concentrations up to 11 mg/L of investigated magnetite nanoparticles coated with aspartic acid, of 9.17 nm average size, in the maize culture medium during germination, showed a positive effect on the growth, chlorophyll biosynthesis and enzymatic activity of catalase and peroxidase. Increased antioxidant enzyme activity suggested an activated defense system by magnetite nanoparticles. Therefore, the low-cost nanoparticle samples may have potential for application as a new nano-fertilizer and could replace the traditional methods of plant growth enhancement. Further studies will be focused on electron microscopy analyses of nanoparticles internalization, activity of several important enzymes and iron level measurements on plant tissues treated with nanoparticles.

## Figures and Tables

**Table 1:** The A-MNP concentration of aqueous diluted A-MNP suspension samples.

A-MNP suspension volume fraction (µL/L)	20	40	80	160	320	400
A-MNP concentration (mg/L)	0.55	1.10	2.20	4.40	8.80	11.00

## Data Availability

All of the data is contained within the article.
